# Multisegmented Nanowires: a Step towards the Control of the Domain Wall Configuration

**DOI:** 10.1038/s41598-017-11902-w

**Published:** 2017-09-14

**Authors:** E. Berganza, M. Jaafar, C. Bran, J. A. Fernández-Roldán, O. Chubykalo-Fesenko, M. Vázquez, A. Asenjo

**Affiliations:** Instituto de Ciencia de Materiales de Madrid, CSIC, Madrid, 28049 Spain

## Abstract

Cylindrical nanowires synthesized by controlled electrodeposition constitute excellent strategic candidates to engineer magnetic domain configurations. In this work, multisegmented CoNi/Ni nanowires are synthesized for tailoring a periodic magnetic structure determined by the balance between magnetocrystalline and magnetostatic energies. High-resolution Transmission Electron Microscopy confirms the segmented growth and the sharp transition between layers. Although both CoNi and Ni segments have similar fcc cubic crystal symmetry, their magnetic configuration is quite different as experimentally revealed by Magnetic Force Microscopy (MFM) imaging. While the Ni segments are single domain with axial magnetization direction, the CoNi segments present two main configurations: a single vortex state or a complex multivortex magnetic configuration, which is further interpreted with the help of micromagnetic simulations. This original outcome is ascribed to the tight competition between anisotropies. The almost monocrystalline fcc structure of the CoNi segments, as revealed by the electron diffraction patterns, which is atypical for its composition, contributes to balance the magnetocrystalline and shape anisotropies. The results of MFM measurements performed under in-plane magnetic field demonstrate that it is possible to switch from the multivortex configuration to a single vortex configuration with low magnetic fields.

## Introduction

The exploration of new strategies to control the domain wall configuration in elongated nanostructures is an active topic of research of significant importance in electronics, nanomedicine and computing. In particular, cylindrical nanowires (NWs) fabricated by controlled electrodeposition into porous alumina templates^1, 2^ are attractive for a variety of technological applications ranging from magnetic recording media^3^, spintronics^4^, sensors^5^, to nanomedicine or biomagnetics^6^. Among other advantages, the low cost fabrication process, the absence of the Walker breakdown that limits the domain wall velocity^7, 8^ and the wide variety of materials and configurations –core shell^9^, multilayered^10, 11^ or modulated in diameter^12, 13^ – make them excellent candidates to engineer magnetic domain configurations.

Multisegmented magnetic/metallic NWs are particularly interesting for engineering new magnetic structures and for the design of advanced spintronic devices^4^. Magnetic/Magnetic multilayers, instead, are designed to control the motion of a propagating domain wall at the frontier of segments with different anisotropy characteristics. Co-based NWs have the advantage of exhibiting a variety of magnetic properties that can be tailored in the fabrication process^14, 15^. Co–Ni alloy NWs are very versatile magnetic materials that can exhibit either a soft or a hard magnetic behavior depending on the Co content. They present a significant magnetocrystalline anisotropy which makes them an alternative to Fe–Ni alloy based systems with small anisotropy^16^. Ni-rich NWs typically exhibit fcc structure, however, when Ni concentration decreases (Ni atoms are partially substituted by Co), a transition from fcc to hcp phase is observed^17, 18^, although both crystalline structures seem to coexist for alloys close to Co_50_Ni_50_^19^.

As stated in previous works^20–22^, CoNi bulk alloys can present very different magnetocrystalline anisotropy values depending on their exact composition. In the case of the NWs, their magnetization easy axis can be tilted from axial to perpendicular by increasing the Co content. As a general rule, compositions closer to pure Co have dominant magnetocrystalline anisotropy, while shape anisotropy determines the magnetization in Ni rich NWs. Thanks to the high solubility of Co atoms in the Ni lattice (and vice versa), it is possible to tailor the stoichiometry.

Although the magnetic properties of arrays of NWs have been studied in different works (see for example refs 17 and 23.), fewer studies have focused on the magnetic states of isolated NWs. The main reason is that the observation of the magnetic configuration in isolated NWs requires advanced techniques with high sensitivity and lateral resolution.

Any practical application of nanowires in spintronics^24, 25^ lies, roughly speaking on the switching of magnetization from one configuration to another. This requires a precise individual characterization of the domain configuration and full understanding of the magnetization reversal mechanism. In this sense, imaging techniques have opened the path to the direct visualization of domain structures.

In comparison to other high resolution techniques as electron holography^26^ or x-ray magnetic circular dichroism photoemission electron microscopy (XMCD-PEEM)^27^, Magnetic Force Microscopy (MFM) is an accessible imaging technique, with magnetic resolution below 20 nm and the possibility to study dynamic processes under *in-situ* applied field.

A high resolution Magnetic Force Microscopy^28^ has been used to determine the domain configuration of the individual NWs^29^. Even more, thanks to the advanced VF-MFM modes^30^ the reversal magnetization process and the magnetostatic coupling between segments can be analyzed.

## Results and Discussion

### Crystallographic structure of CoNi segments

The objective of this investigation has been to design and characterize isolated multisegmented NWs with tailored magnetic anisotropy leading to specific magnetic domain structure and response under applied magnetic fields.

Multisegmented CoNi/Ni cylindrical NWs 120 nm in diameter and 20 μm long have been grown by electrochemical route into anodic alumina membranes (AAM) for the specific purpose of the present investigation. Ni and CoNi alloy segments have been alternated in the fabrication of these NWs (see Methods Section for more information). Their lengths, later confirmed by structural analysis, were chosen to conform a net of rods with low magnetic interaction between segments. Ni was chosen as a spacer between CoNi segments, due to its low magnetization (0.61 T). In addition, its lattice period allows good matching with CoNi alloy.

On the other side, their different magnetocrystalline anisotropy values promote the development of different magnetic configurations. While in Ni segments the shape anisotropy dominates, in CoNi regions a competition between magnetocrystalline and shape anisotropies is expected^31^. Moreover, recently D. Reyes *et al*.^32^. highlighted the strong dependence of the magnetic features of the NWs on their structural properties.

**Figure 1:**
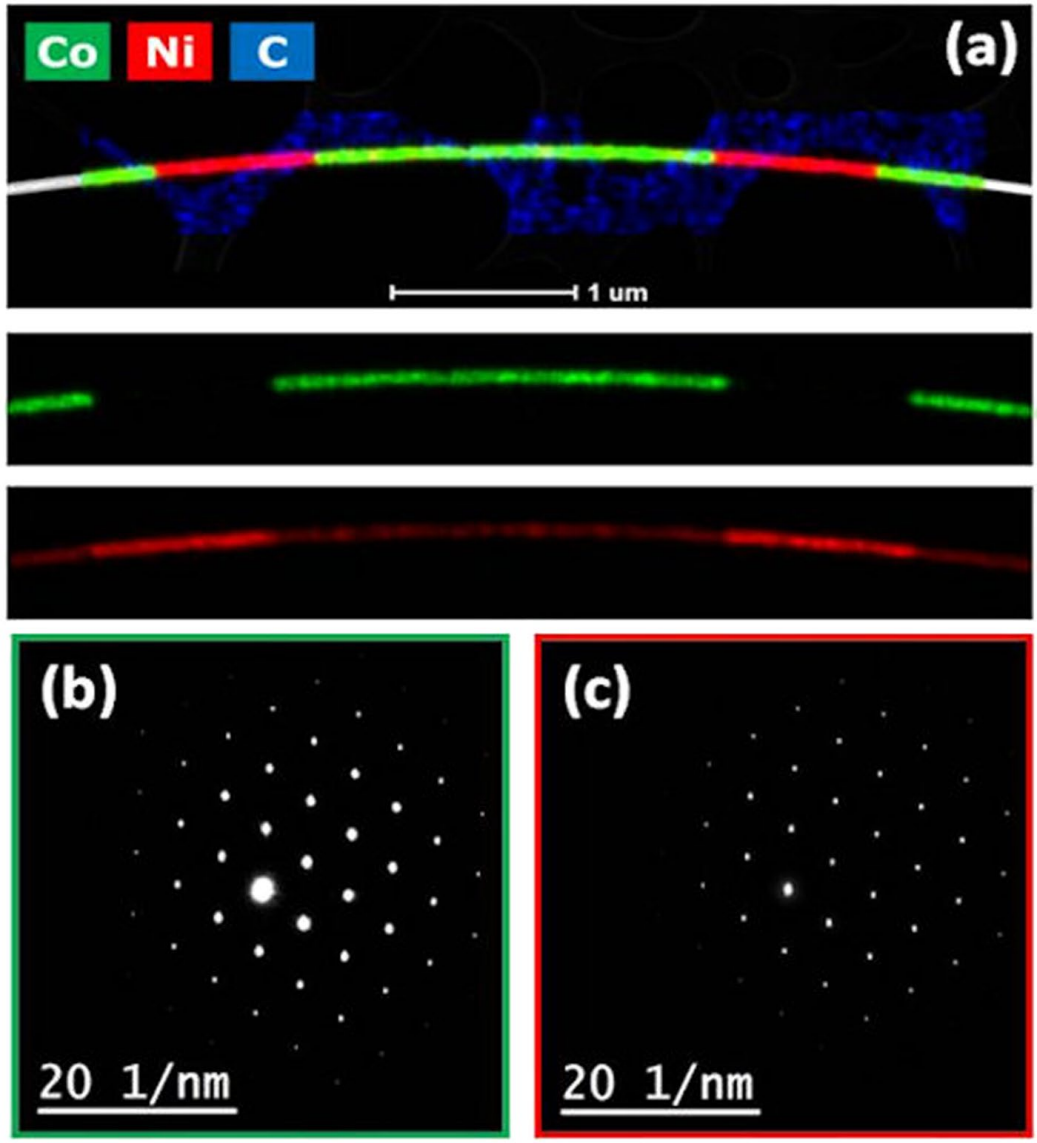
(**a**) Chemical mapping though XEDS allows identification of Co and Ni regions. STEM data superimposed to XEDS mapping as well as the separately XEDS maps corresponding to Co and Ni are shown. In (**b**) and (**c**) electron diffraction patterns of Co and Ni are presented, respectively. Both Co and Ni grow in the direction as shown.

To establish the relationship between structural and magnetic features, we carry on energy-dispersive X-ray spectroscopy (XEDS) measurements that enable us to map the regions with different composition and quantify the stoichiometry of the CoNi alloy (see Fig. 1). The Co_65_Ni_35_ segment composition has been determined and it was confirmed to be constant along the nanowire. XEDS mapping verify the sharp transition between the two layers, with an intermixing area around 15 nm for Co into Ni layer (see Fig. S1, Supplementary information).

Selected area electron diffraction (SAED) patterns confirms that Ni segments grow along the <110> direction with fcc structure.

Moreover, although it is expected that this Co_65_Ni_35_ alloy presents a mixture between fcc and hcp phases^19^, CoNi segments grow epitaxially onto Ni fcc layer with a lattice parameter less than 1% bigger than the Ni lattice value. This means that in both layers the angle between the magnetic easy axis <111> and the wire axis is 35°. As deduced from the STEM data, the size of the CoNi and Ni crystals are above 400 nm.

### Single and multivortex domain configurations

**Figure 2:**
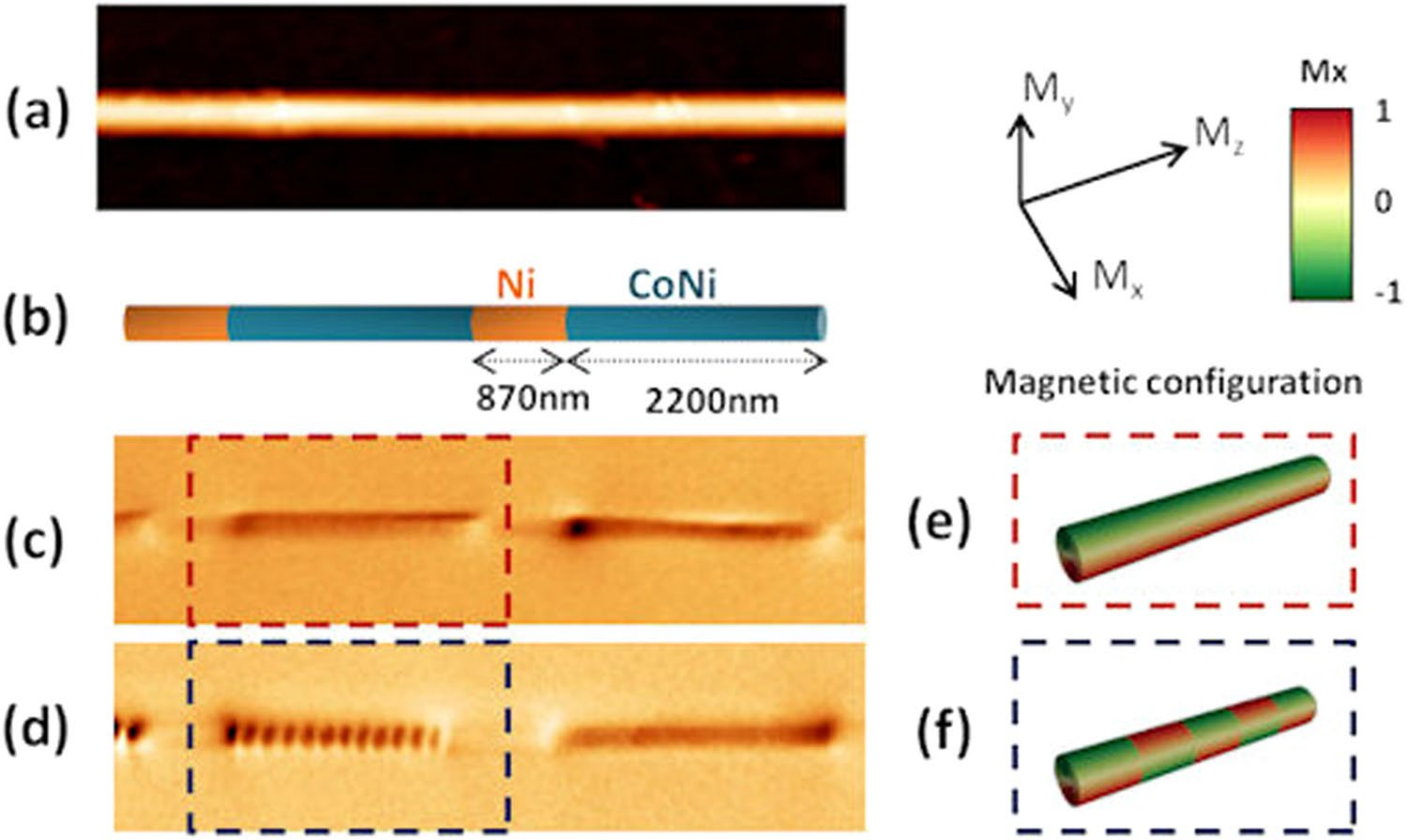
(**a**) Topography, (**b**) geometry sketch and (**c**) and (**d**) MFM images of different NWs in as prepared magnetic state. Two different behaviours are shown by different CoNi segments. In (**c**) the segment in the red square shows a uniform magnetization, while in (**d**) a multidomain structure is displayed. (**e**) and (**f**) schematically show the expected magnetization configurations corresponding to (**c**) and (**d**) MFM images, respectively.

MFM imaging in the conventional lift mode at a constant height (see Methods) was performed to investigate the domain configuration of individual NWs. Figure 2 presents the results that correspond to the as prepared magnetic state of a piece of NW (an entire wire is shown in Supplementary Information, Fig. S2). Different features can be clearly distinguished. Firstly, the difference observed between the Ni and CoNi segments in the MFM signal: although in the topographic image (Fig. 2a) both segments look identical, the MFM images (Fig. 2c,d) reveal different magnetic configurations. On the one hand, all the Ni segments display a weaker and uniform contrast along the entire segment and, in most of the cases, a bright-dark dipolar contrast appears at the ends of the Ni segment. These MFM results are in good agreement with the single domain configuration expected for the Ni segments where the shape anisotropy, one order of magnitude higher than the magnetocrystalline anisotropy, determines the magnetic configuration. On the other hand, contrary to the Ni segments, after performing statistics of more than 20 NWs and 80 segments, we found that the CoNi segments show two different trends. Some NWs display a dark/bright quasi-homogeneous contrast along the segment (see region marked with a red dashed square in Fig. 2c). The other trend is shown in Fig. 2d, where the magnetization of the CoNi segment breaks into domains (see region marked with a blue dashed square). Notice that due to the cylindrical shape and the size of the diameter, 120 nm, the vortex configuration (with one or more vortices along the NW length) is expected for Co based NWs^12^. Moreover, both states have to be compatible with the magnetization easy axis parallel to the NWs as measured by VSM (See Fig. S3, Supplementary Information) and should be a consequence of the competing balance between magnetostatic energy (in the shape anisotropy approximation K_sh_ = 157.5 kJm^−3^) and cubic magnetocrystalline anisotropy K_1_ = 144 kJm^−3^ for a single CoNi segment. These values are very close and produce a soft magnetic behavior, thus favoring the formation of vortices. Considering all this, MFM images from CoNi segments are interpreted as a single vortex with an axial core (Fig. 2e) or a sequence of vortices of opposite chiralities (Fig. 2f).

**Figure 3:**
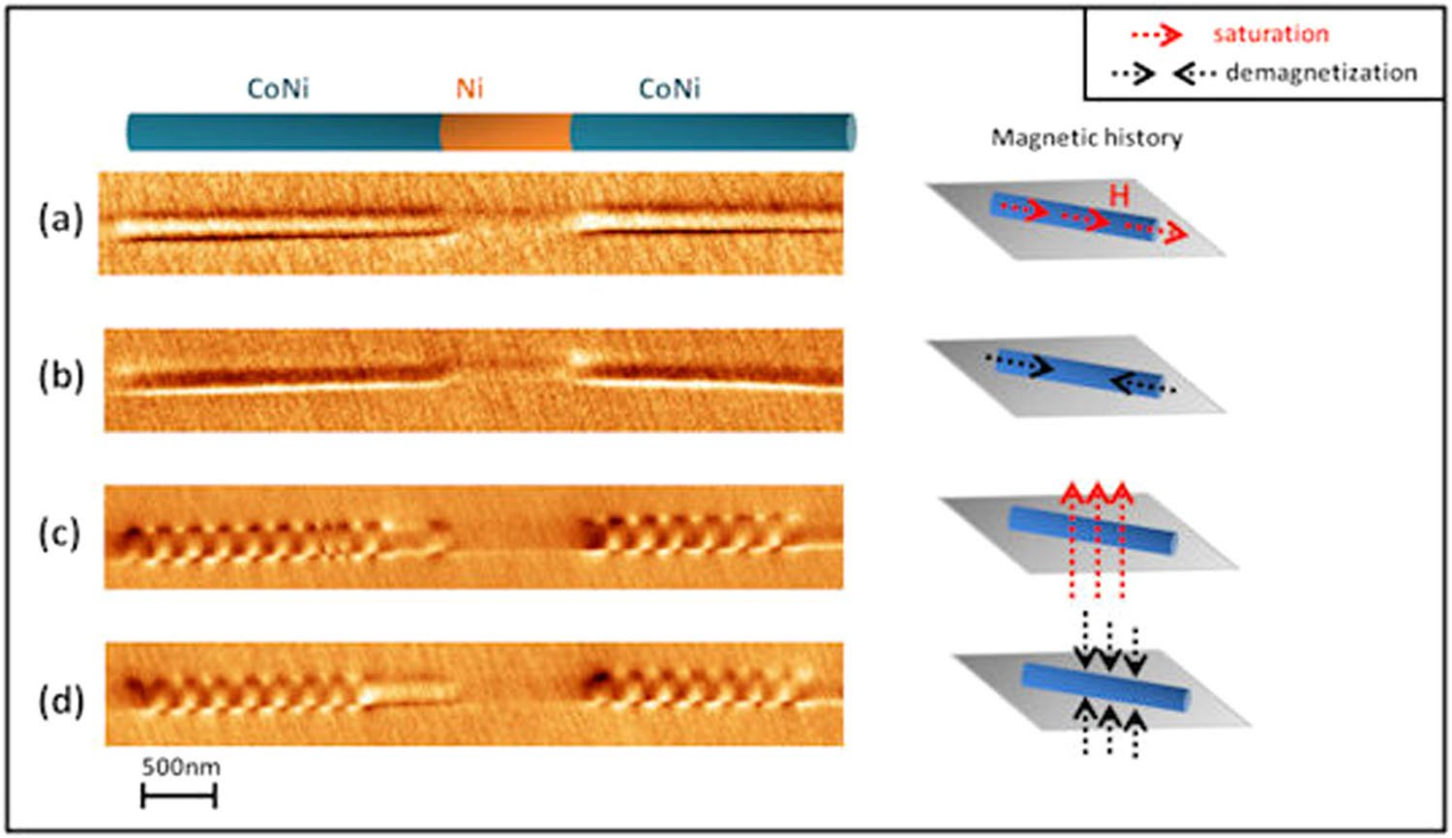
(**a**) Series of MFM images in a remnant state corresponding to the same piece of nanowire with different magnetic history after (**a**) applying a magnetic field of 1.8 T in the axial direction, (**b**) demagnetizing in the axial direction, (**c**) applying a perpendicular magnetic field of 1.8 T, and (**d**) demagnetizing in perpendicular direction.

The tight anisotropy balance of CoNi segments makes them suitable candidates to switch from one configuration to another by applying external magnetic fields. In Fig. 3, the same piece of NW is successively exposed to magnetic fields of a maximum amplitude of 1.8 T and the remnant magnetic configuration is imaged. The region displayed in the MFM images in Fig. 3 corresponds to two CoNi segments separated by a Ni layer. Notice that the contrast in Ni segments keeps constant when the magnetic field changes, although the CoNi segments can develop a complex configuration.

After applying a saturating field (Fig. 3a) or demagnetizing sequence (Fig. 3b, where the field switches from positive to negative values with a decreasing amplitude value) along the axial direction, a uniform contrast is observed in the MFM images. In addition, a dark/bright contrast appears in the lateral side of the segment. This configuration is similar to the one in Fig. 2c,e. However, after applying a saturating magnetic field perpendicular to the nanowire axis, *i*.*e*. perpendicular to the magnetization easy axis, a multidomain periodic structure is again obtained in the MFM images (Fig. 3c). Such zig-zag configuration with an alternate bright/dark contrast is analogous to the one shown in Fig. 2d,f. Similar behaviour is found in the MFM image in Fig. 3d obtained after exposing the NW to a perpendicular demagnetizing sequence. This observation allows us to determine the reproducibility of this complex structure.

**Figure 4:**
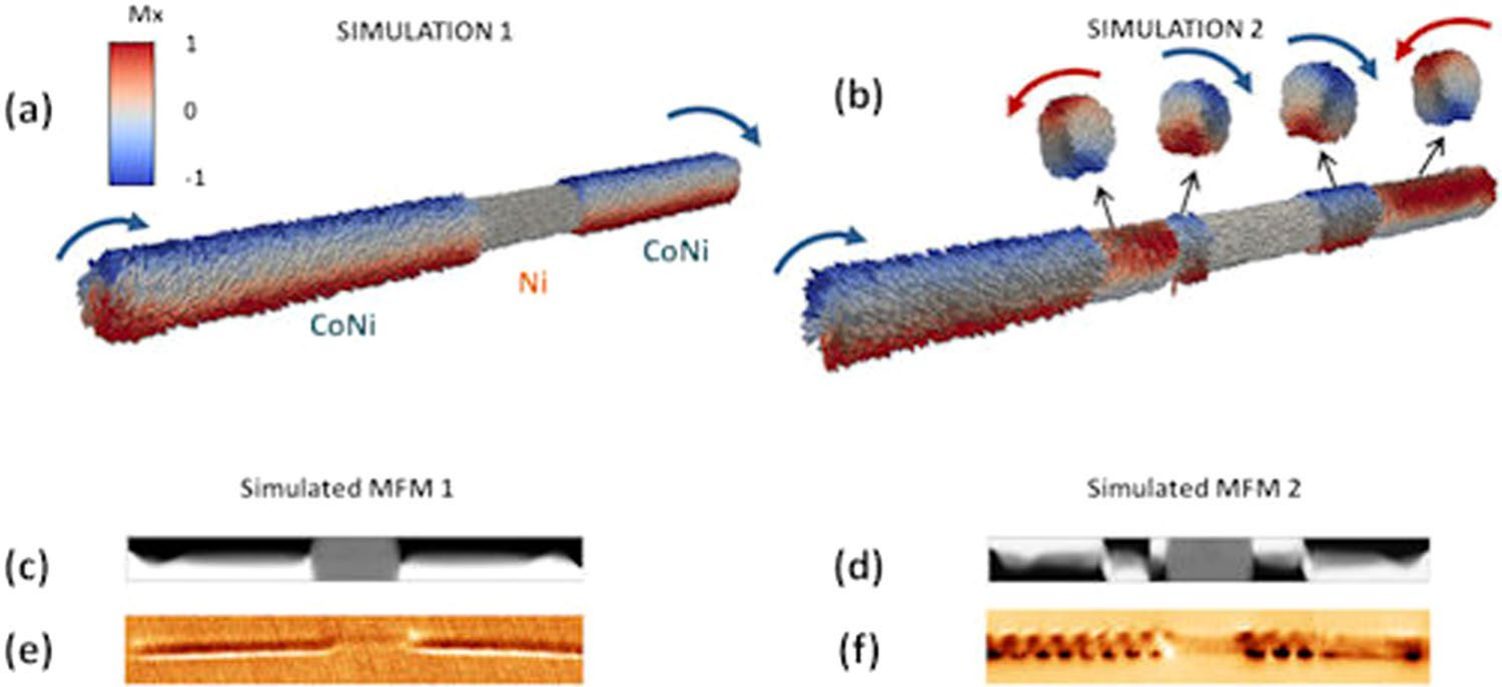
Micromagnetic simulations show (**a**) a vortex along the two CoNi segments and a Ni segment with axial magnetization and (**b**) multivortex configurations in CoNi segments and a Ni segment with axial magnetization. In both Figures, positive and negative Mx components of the magnetization are depicted in red and blue colours and the grey colour is used for almost zero value. (**c**) and (**d**) display the corresponding simulated MFM images of the configurations shown in (**a**) and (**b**). Experimental MFM images displaying: (**e**) the same configuration as in (**c**) and (**f**) configuration similar to the one shown in (**d**).

The complex configuration in cylindrical NWs with magnetization easy axis along the nanowire axis, sparks new questions. Micromagnetic simulations are performed to shed light into the origin of this novel configuration and the coexistence of single and multivortex configurations, like the ones shown in Fig. 3b,d. A three-layer CoNi/Ni/CoNi element has been considered for the following two simulations: In simulation 1, the initial magnetic configuration of the system was chosen as single vortex with the same chirality for each CoNi segment and axial magnetization for the Ni layer. The system was evolved until the minimum energy state was reached. The resulting configuration is shown in Fig. 4a. In simulation 2, the remnant state configuration is simulated by gradually decreasing the perpendicular applied field from 1.8 T to 0 T. Parameters and further details are found in the Methods Section.

Results show that Ni segments present always a simple axial magnetization. On the other hand, the single vortex spin configuration obtained for each CoNi segments after relaxation is a minimum energy state (Fig. 4a). Simulation 2 shows a multivortex structure with consecutive vortices of opposite chiralities (Fig. 4b) in the remnant state. Notice that the magnetic configurations of the CoNi segments are independent of the adjacent CoNi segments. The results are consistent with the configuration obtained experimentally, where the pattern of vortices is, nevertheless, periodic.

The simulations confirm that, in remanence, the magnetization of the CoNi core and Ni segments are parallel to the main axis. However, the CoNi regions present a complex magnetic configuration that tends to develop a vortex or multivortex states, as shown by the cross sections in Fig. 4b for each vortex. It has been seen that the core of these vortices is a little bit displaced from the axis of the NW and the vortices themselves are tilted towards the anisotropy axis. Another interesting feature unveiled by the simulation is the possibility of the existence of superficial vortices.

Figure 4c,d display two simulated MFM images calculated from the configuration shown in Fig. 4b, considering that MFM is sensitive to the magnetization gradient. In the Supplementary Information, Fig. S4, more details on small differences that might come up when measuring the multivortex configuration in MFM are given.

According to the energy values obtained with micromagnetic simulations, the multivortex state has nearly twice as much energy as the single vortex state. Despite being a single vortex energetically more favourable and therefore more common, either state is possible and can coexist in the same segment, as it has been experimentally proven (Fig. 3d). This indicates that although the multivortex configuration represents a metastable state, it can be accessed by changing the magnetic history or stabilized by the defects.

Further simulations have been carried out to determine the role of the multilayer in the overall magnetic behaviour of the system. Simulations unveil that the size of the CoNi segments plays a role on the stability of the multivortex structure. Besides, for single vortex configurations in CoNi, the most likely combinations of chiralities and polarities have been studied (see Supplementary Information, Figs S5 and S6).

### Study of domain configuration and coupling between segments under *in-situ* applied field

**Figure 5:**
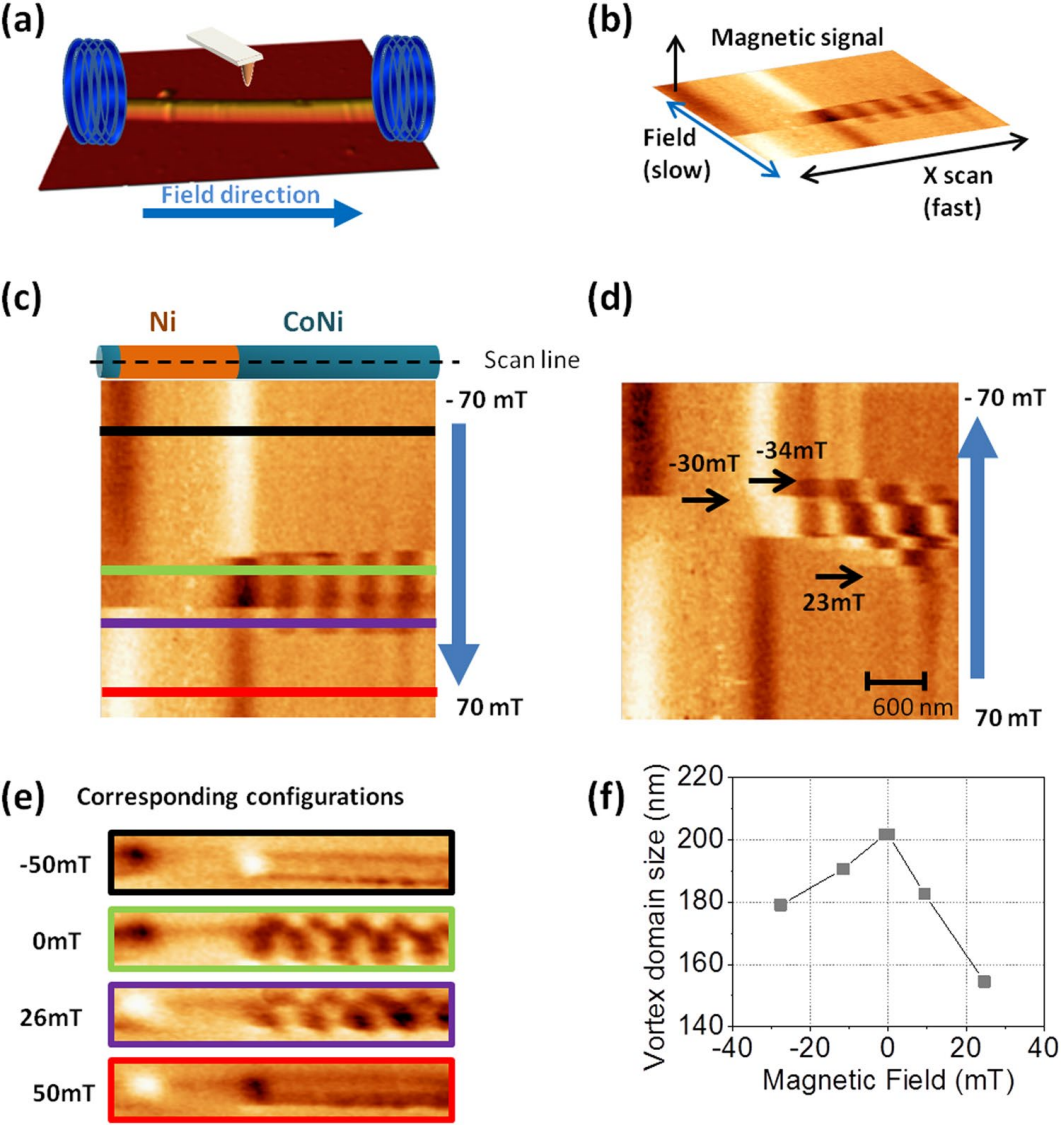
(**a**) The sketch shows the layout of the system. (**b**) Explains images obtained by the non- standard advanced MFM operation mode where the magnetic field parallel to the NW axis is swept between two values while the magnetic signal is recorded along the central line over the NW. CoNi-Ni-CoNi segments are imaged by non-standard advanced MFM showing different behaviour under applied field. (**c**) and (**d**) represent two branches of the evolution of the magnetization of the Ni and CoNi segments. The profiles marked in (**c**) correspond to the configuration measured in the MFM images obtained at that magnetic fields presented in (**e**). (**f**) Vortex domain size decreases with the positive or negative increasing field magnitude. Data extracted from images (**c**) and (**d**).

Finally, further measurements have been carried out with *in situ* applied magnetic field along the NWs using advanced MFM operation mode explained elsewhere^30, 33^. A piece of NW with a multivortex state is chosen to track its evolution under applied magnetic fields (Fig. 5). X scan is taken parallel to the NW axis and the top of the NW is repeatedly scanned along the dotted black line (see Fig. 5a and sketch in Fig. 5c). Therefore, slow scan (Y axis) represents the magnetic field sweeping between maximum/minimum values of ±70 mT (Fig. 5b). Two branches are shown in Fig. 5c,d thus, a minor hysteresis loop is imaged. As it can be inferred, for the higher field values the multivortex configuration disappears and the configuration changes to a single vortex state with the core oriented with the field (black and red lines with their corresponding images at fixed values of the field, Fig. 5c,e). This imaging mode allows us to quantify the critical fields for each configuration or reconstruct a hysteresis loop of a segment (See Supplementary Information, Fig. S7).

We observe that the multivortex configuration only exists for a narrow range of values, namely between 20 and 35 mT (red and purple lines with its corresponding images in Fig. 5d,e). Although the information obtained by MFM is mainly superficial, these intermediate stages give us an idea of how the magnetization reversal takes place. In the second branch (Fig. 5d), at 23 mT, a couple of vortices of opposite chiralities nucleate in the CoNi segment. At a slightly higher field amplitude, more vortices nucleate and move along the NW. Numerical data (Fig. 5f) prove that vortex domains tend to narrow when the NWs are subjected to either positive or negative increasing fields.

In fact, when field is applied, the growth of one (or more) vortex domain is favoured at the expense of the rest, whose size decrease until they are annihilated. We need to keep in mind that CoNi segments have their magnetization easy axis tilted 35° from the NW axis, which makes the cores a bit slated from the axis, and therefore, their outer magnetization have a little axial component. This fact, together with the magnetic charges of the Ni segments, might energetically favour one particular vortex to extend while the rest decrease their size.

Another remarkable feature is that the magnetization reversal process is not fully symmetrical, since more intermediate configurations are found in the second branch than in the first one. However, in the first branch (Fig. 5d), additional intermediate configurations might exist but presumably, some changes occur at a speed that our data acquisition timings do not make their imaging possible.

Further details are given in Supplementary Information Fig. S8, where VF-MFM images reveal that the polarity of the CoNi segment reverses without affecting the multivortex configuration.

## Conclusions

Multisegmented NWs with alternating magnetic layers were designed and grown. Two magnetic materials CoNi and Ni have been chosen, in such a way that the CoNi alloy grows epitaxially onto the Ni lattice, with a very sharp interface. HR-TEM reveals that, Co_65_Ni_35_ segments grow in fcc texture, unlike the CoNi nanowire alloys of intermediate composition where hcp and fcc coexist. This feature gives rise to a fcc anisotropy in CoNi capable to compete with the magnetostatic energy. As a consequence, two possible structures are developed: a single vortex configuration and a periodic multivortex structure with opposite vortex chiralities. As we demonstrate by using high resolution Magnetic Force Microscopy, we can go from one state to the other by applying rather low external magnetic fields.

Since the switching of the core is independent of the vortex chirality, in future works, the polarity and chirality could be independently tailored, for the development of devices for storage information or sensor devices.

## Methods

### Sample Fabrication

[CoNi/Ni]10 NWs were prepared by filling the pores of anodic aluminum oxide (AAO) templates by electroplating.

The templates were obtained by hard anodization in oxalic aqueous solution (0.3 M) containing 5 vol.% ethanol at a constant temperature of 0–1 °C^34^.

Nanopores with 120 nm in diameter and 60 µm in length were obtained. Afterwards, the residual Al and the alumina barrier layer at the bottom of the foils were chemically etched, and an Au layer was sputtered to serve later as an electrode for final electroplating of NWs. The multisegmented NWs were grown into the nanopores of AAO templates, at room-temperature, by DC electrodeposition using two different electrolytes: NiSO_4_ (0.75 M) + NiCl_2_ (0.3 M) + H_3_BO_3_ (0.3 M) and CoSO_4_ (0.125 M) + CoC_l2_ (0.08 M) + NiSO_4_ (0.065 M) + NiCl_2_ (0.11 M) + H_3_BO_3_ (0.32 M). Both segments were electrodeposited at a constant voltage of 1.2 V for different periods of time, 20 s for Ni and 80 s respectively, for CoNi segment. The bilayers were repeated 10 times.

Finally, the alumina was dissolved by using a mixed solution of CrO_3_ and H_3_PO_4_. After repeatedly wash of individual NWs they were dispersed on a Si substrate by spin coating.

### Transmision electron microscopy

A FEI Titan Themis 60–300 kV system was used for the structural characterization, operated at 200 kV. It is equipped with a double aberration-corrector hardware, a high efficiency EDX signal collector and a CMOS camera of 4kx4k pixels. The crystalline structure has been studied for different NWs and areas.

### Magnetic force microscopy

A scanning force microscope of Nanotec Electronica was used for the MFM measurements. The amplitude modulation mode together with the Phase Locked Loop (PLL) enabled (to make the phase zero and keep the maximum amplitude) was selected. Homemade Co-MFM probes where used, as well as commercial probes from Budget Sensors MagneticMulti75-G, with CoCr coating.

### Micromagnetic simulations

Micromagnetic simulations were performed using an object oriented micromagnetic modeling framework (OOMMF)^35^. Two segments of 1 µm long Co_65_Ni_35_ separated by a Ni segment of 500 nm were considered. The diameter is 120 nm and we have considered the fcc texture, grown in the <110> direction as measured by HR TEM. The material parameters used for each composition are shown in Table 1. The magnetocrystalline anisotropy of Ni is low compared with the magnetostatic energy density, and thus, is set to zero.

**Table 1:** Parameters of the different materials used for the micromagnetic simulations.

Material	*M*_*S*_ [A m^−1^]	*A*_*ex*_[J m^−1^]	*K*_1_[J m^−3^]	Anisotropy
Ni	0.48·10^6^	0.34 10^−11^	0	none
Co_65_Ni_35_	1.08·10^6^	0.45·10^−11^	6.4·10^4^	cubic

Saturation magnetization was weighted regarding the stoichiometric composition with the values found in the literature. Saturation magnetization Ms was set 1.08∙10^6^ A m^−1^, exchange stiffness A_ex_ = 0.45∙10^−11^ J m^−1^ (see ref. 36) and 0.34∙10–11 J m^−1^ and anisotropy constant K_1_ = 6.3∙10^4^ J m^−3^ (see ref. 19) and K_1_ = 0 for the CoNi and Ni segments respectively. Ni parameters have been chosen according to ref. 37.

## Electronic supplementary material

Supplementary Information
