# Supplementary material for: Multisegmented Nanowires: a Step towards the Control of the Domain Wall Configuration
Source: Sci Rep. 2017 Sep 14;7:11576. doi: 10.1038/s41598-017-11902-w (PMC5599633; doi:10.1038/s41598-017-11902-w)
Supplement: Supplementary file 1 — Supplementary Information [file 41598_2017_11902_MOESM1_ESM.doc]

# Multisegmented Nanowires: a Step towards the Control of the Domain Wall Configuration

E. Berganza^1^, M. Jaafar^1^*, C.Bran^1^, J. A. Fernández-Roldán^1^, O. Chubykalo-Fesenko^1^, M. Vázquez^1^ and A. Asenjo^1^

^1^Instituto de Ciencia de Materiales de Madrid, CSIC, Madrid, 28049, Spain

Supplementary information

1. Interface between layers

The XEDS profile along the interface shows a sharp transition between the CoNi and Ni layers, with an intermixing area below 15nm.

**Fig. S1.** (a) XEDS profile along the interface between layers, zoomed in (b). The piece of NW is shown in (c), with compositional contrast.

1. MFM image of a complete NW

**Fig. S2.** The image of a full NW has been reconstructed, from 3 MFM images. The wire was imaged in the –as prepared- magnetic state.

1. Macroscopic magnetic behavior

VSM Hysteresis loops performed of the NWs in the membrane. As it is indicated in the image, they have been performed both in the axial and perpendicular directions. It is deduced from the macroscopic loops that the easy axis lies along the NW axis.


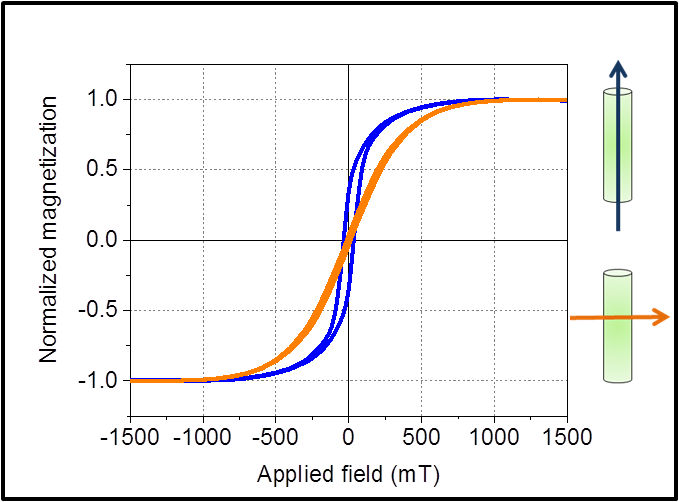


**Fig. S3.** Normalized hysteresis loop of the NWs inside the membrane.

1. Relative orientation of the easy axis of magnetization on the substrate


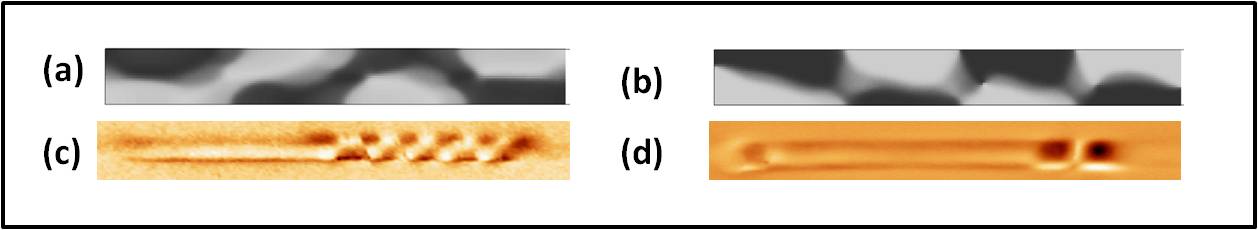


**Fig. S4. (a)** and **(b)** display simulated MFM images of the same CoNi segment with different orientation of the nanowire easy axis with respect to the substrate. **(c)** and **(d)** are examples of experimental MFM images of CoNi segments that present magnetic configurations similar to the ones simulated in (a) and (b)

The MFM images of multivortex segments might look different depending on the orientation of the nanowire, and its easy axis with respect to the substrate.

As shown in Fig. S4a and Fig. S4b, nanowires with the same magnetic configuration but different crystalline orientation with respect to the substrate can display different MFM images. We have to keep in mind that the anisotropy easy axis of the NWs- <111> directions for fcc is tilted 35º with respect to the cylinder axis. This means that since there is no axial symmetry, the MFM image might be slightly different depending on the orientation of the easy axis of the NW with respect to the substrate. This is something that is statistically expected for the experimental MFM measurements. See, for instance, the differences between Fig. 2d and Fig. 3b.

1. Magnetic effect of Ni

Single vortex configuration might display four states combining positive and negative chiralities (Q) and polarities (P). For the subsequent simulations, we follow the minimum energy configuration criteria for a three layer CoNi/Ni/CoNi NW with chosen initial magnetic configuration. As shown in Fig. S5a and S5b, single vortices were initially chosen for the two CoNi segments with opposite chiralities (Fig S5a) or polarities (Fig.S5b).

The corresponding minimum energy configurations are shown in Fig. S5c and S5d respectively. Since the vortices are not completely axially symmetric, we can expect some influence of the Ni segment magnetization on the vortex chirality in CoNi. However, in our simulations the presence of Ni segments does not seem to determine the chirality of the single vortices states in CoNi segments, as all the configurations (Q_1_=Q_2_ and Q_1_=-Q_2_) correspond to the energy minima. In the case of polarity, though, Fig. S5d shows that when opposite polarities are chosen as initial configuration (Fig.S5b), one of the CoNi segments inverts the polarity to minimize the energy.

As proven by the third simulation (Fig. S5f) the CoNi segments are far enough to be non-interacting, and therefore, show independent polarities if the Ni layer is substituted by a non-magnetic spacer.


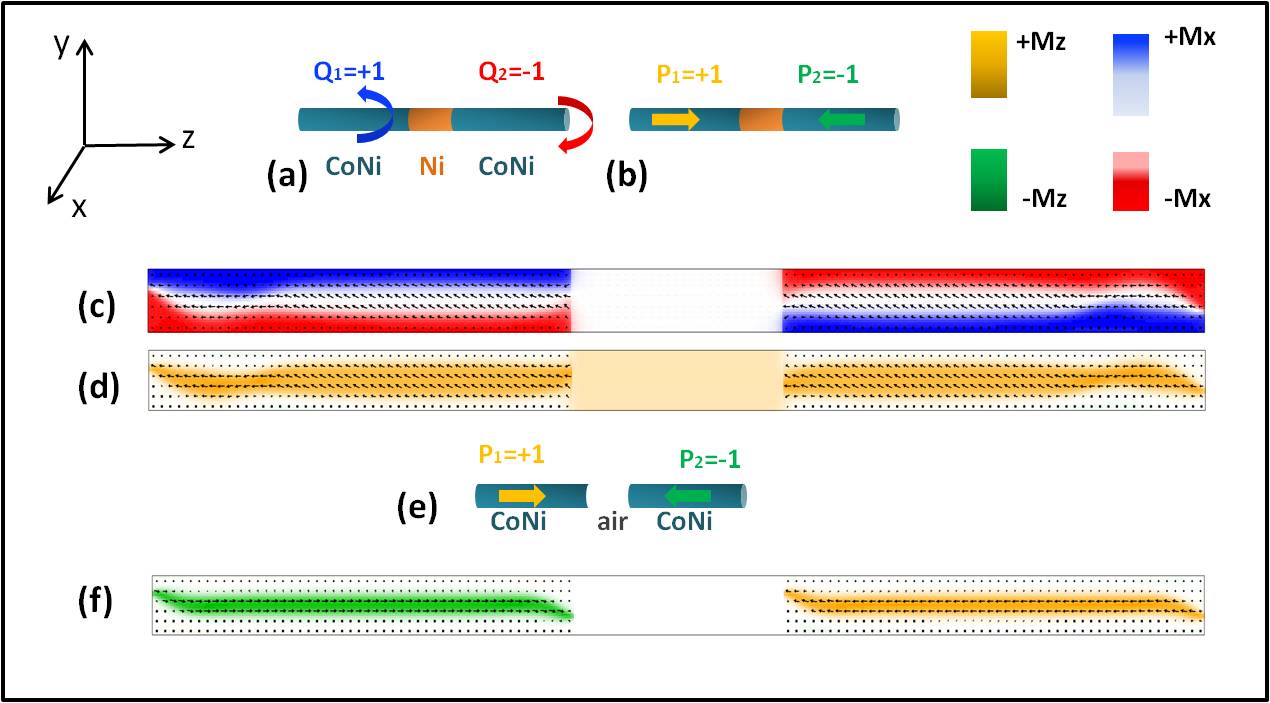


**Fig. S5. (a)**, **(b)** and **(e)** show the sketch of the initial configuration. **(c)**, **(d)** and **(f)** display the resulting minimum energy configurations.

Whether Ni segments favor the formation or not of multivortex configuration is still an open question. Also the dimension of CoNi could play a role on the formation, stability and periodicity of the multivortex structure. Three preliminary simulations were performed to shed some light on this question. For all of them, an initial configuration of multivortices was chosen for CoNi, with a period of 250nm. In Fig.S6a, a 2.5 µm continuous NW was simulated. In the resulting configuration, only one out of 10 vortices (Fig. S6b) did not annihilate. If the length of the segment is decreased to 1 µm the 4 vortices of the initial configuration remain. Next, a three-layer piece of NW (CoNi/Ni/CoNi, Fig. S6c) was simulated to study the effect of the presence of the Ni segment. As shown in Fig. S6d, the presence of the Ni segment lead to annihilation of the multivortex structure in favor of a double vortex state. Notice that in each segment, only 2 out of 4 initial vortices remain. Finally, if the Ni is substituted by a non-magnetic spacer (Fig. S6e), the initial multivortex structure remains (Fig. S6f).


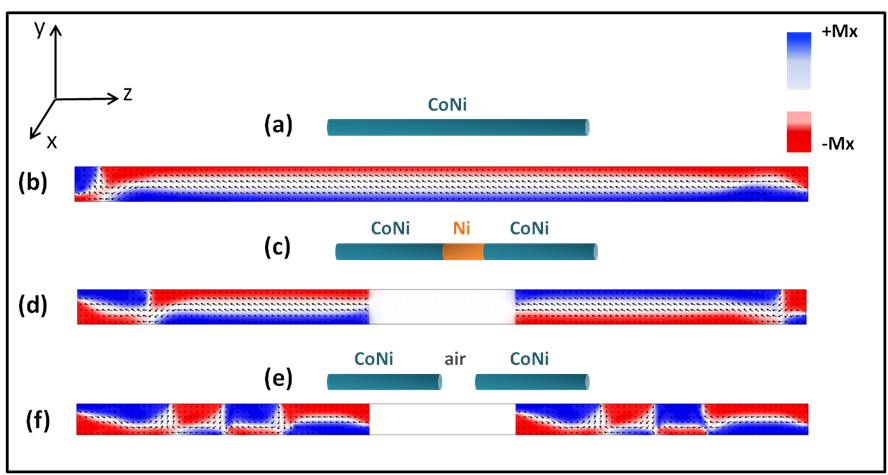


**Fig. S6.** **(a)**, **(c)** and **(e)** represent the type of simulated nanowires chosen for each simulation, where the initial magnetization is always multivortex for the CoNi segments. **(b)**, **(d)**, and **(f)** show their corresponding minimum energy configurations.

1. Hysteresis loop of a Ni segment

A vertical profile along the position of the interface between Ni and CoNi, as marked in Fig. S7a and S7b, allows us to reconstruct the hysteresis loop of the Ni segment (Fig. S7c). From this profiles we can measure the coercive field for this particular Ni segment which is nearly 30mT.

**
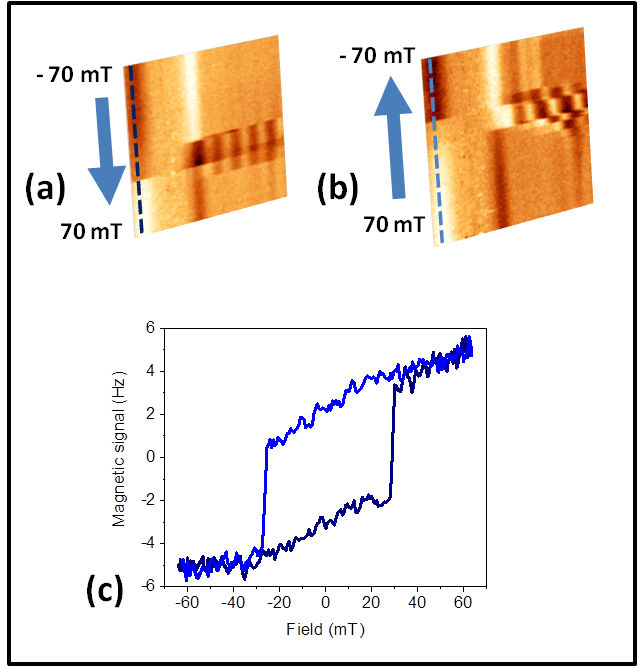
**

**Fig. S7. (a)** and **(b)** Nonstandard MFM images where the evolution of the magnetic contrast as a function of the magnetic field is measured. **(c)** Magnetic loop corresponding to the reversal magnetization process of the Ni segment obtained from the profiles marked with a dashed blue line in **(a)** and **(b).**

**VII- Evolution under in-situ field**

In the images below a CoNi-Ni-CoNi segment is subjected to an axial field. Notice that the right part is the same piece of NW measured by non-standard MFM in Fig. 5. Negative fields are applied to detect the configurations changes unveiled by Fig. 5d. The field step is small, in order to detect the subtlest modifications.

At first glance, we can observe that the two CoNi segments behave differently (Fig. S8 a-k). This might arise due to the fact that the two segments are not completely aligned with the applied magnetic field. Note that in Fig. S8 l-u, subsequent images at decreasing fields have been subtracted to emphasis the differences between them. This method allows us to very accurately determine the changes ongoing from one configuration to the next.

Notice that in the segment on the right, the multivortex configuration remains when the field amplitude is increased. Most remarkably, from configuration S8f to S8g, the axial component of the NW is switched, while no major changes occur in the multivortex configuration displayed by CoNi. The imaging shows direct evidence of the fact that polarity and chirality act independently in this complex magnetic system. Moreover, images S8l, S8o and S8q show evidence of the very subtle changes in the vortex domain size discussed in the main text.

On another note, the CoNi segment on the left, hardly develops a multivortex structure (except at the first stages as shown in Fig. S8l and S8m): when the field amplitude is increased, a single vortex state is achieved. Nevertheless, it reaches a double vortex structure which does not vanish under the fields we have available.


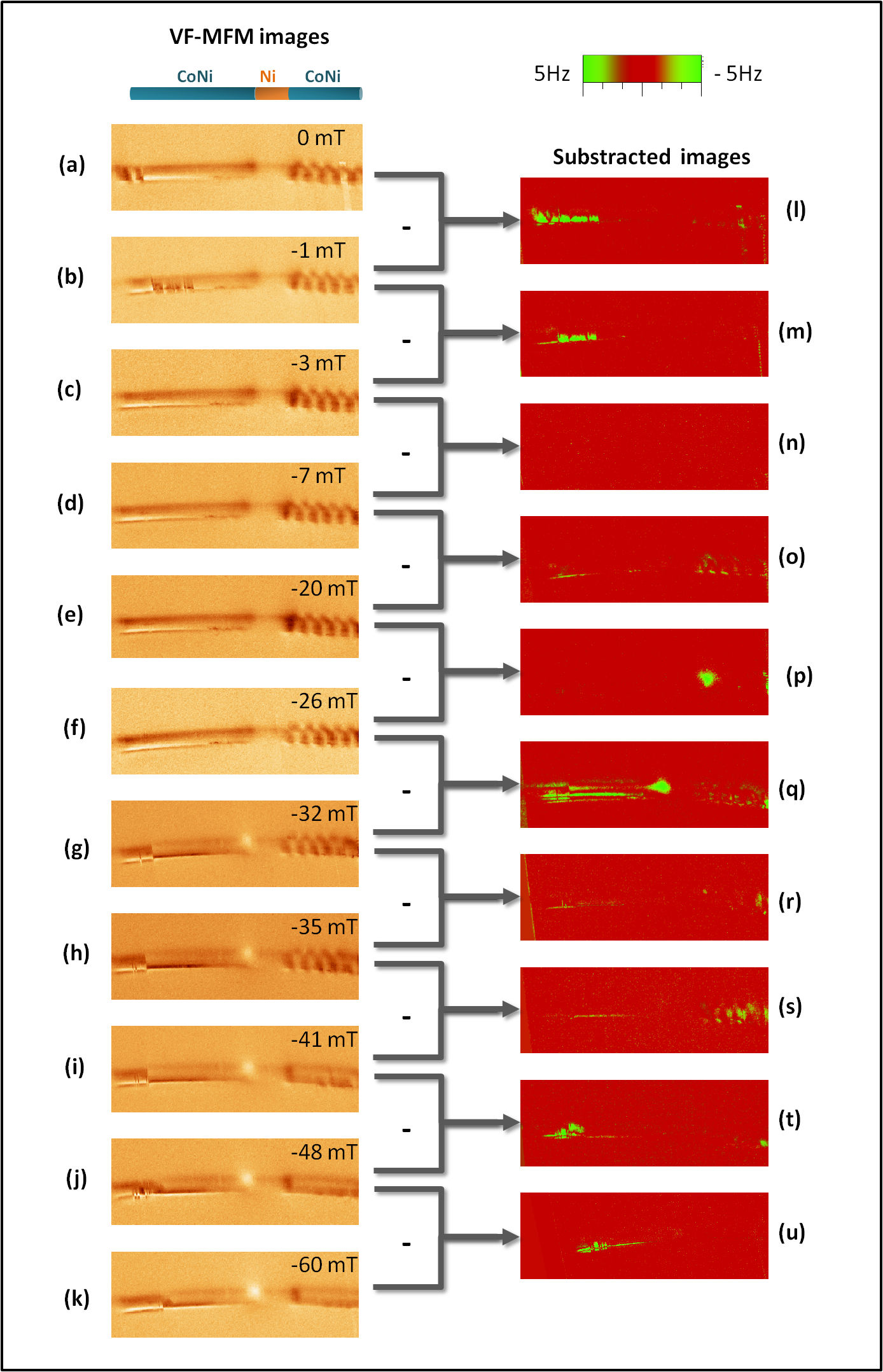


**Fig. S8. (a)** - **(k)** present MFM images under in-situ axial applied fields. **(l) – (u)** are resulting subtracted images of one MFM configuration and the previous one in the sequence.
